# Supplementary material for: Mutation in glutamate transporter homologue GltTk provides insights into pathologic mechanism of episodic ataxia 6
Source: Nat Commun. 2023 Mar 31;14:1799. doi: 10.1038/s41467-023-37503-y (PMC10066184; doi:10.1038/s41467-023-37503-y)
Supplement: Supplementary file 1 — Supplementary Information [file 41467_2023_37503_MOESM1_ESM.pdf]

Mutation in glutamate transporter homologue GltTk provides insights into pathologic mechanism of episodic ataxia 6

Emanuela Colucci[1], Zaid R. Anshari[1], Miyer F. Patino-Ruiz[1], Mariia Nemchinova[1], Jacob Whittaker[1], Dirk J. Slotboom[1*] & Albert Guskov[1*]

[1]Groningen Institute for Biomolecular Sciences and Biotechnology, University of Groningen, 9747AG Groningen, The Netherlands

*Correspondence to d.j.slotboom@rug.nl and a.guskov@rug.nl

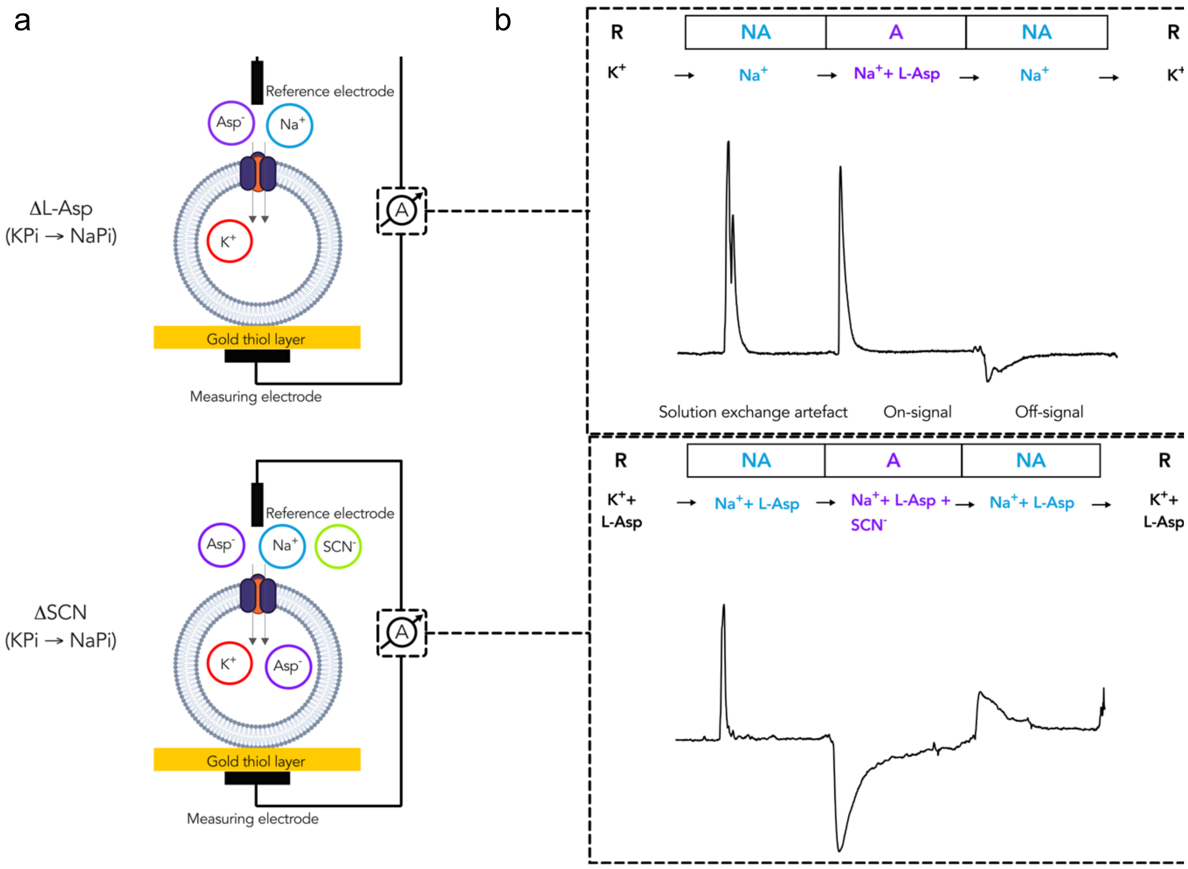

Supplementary figure 1. Schematic representation of the SSM-based electrophysiology method and the signal of the transient current obtained from a double-solution exchange experiment for the L-Asp (top) and SCN^- transport (bottom). (a) The proteoliposomes get absorbed onto the artificial bilayer supported on the gold sensor and the exchange of the solutions generates an electrogenic transport that charges the proteoliposomal membrane which charges the measuring electrode. The measuring principle is called capacitive coupling. Then, charging of the membranes leads to a current flow in the external measuring circuit which is recorded by the amplifier as a transient current. (b) The resting solution [R], that has the same composition of the intraliposomal solution, is exchanged with the non-activating solution [NA] that generates an artefact. The on-signal is the transient current generated by the exchange of the non-activating solution with the activating solution [A], followed by the off-signal given by the exchange with the non-activating solution. After that the initial conditions are re-established by exchanging the non-activating with the resting solution [R].

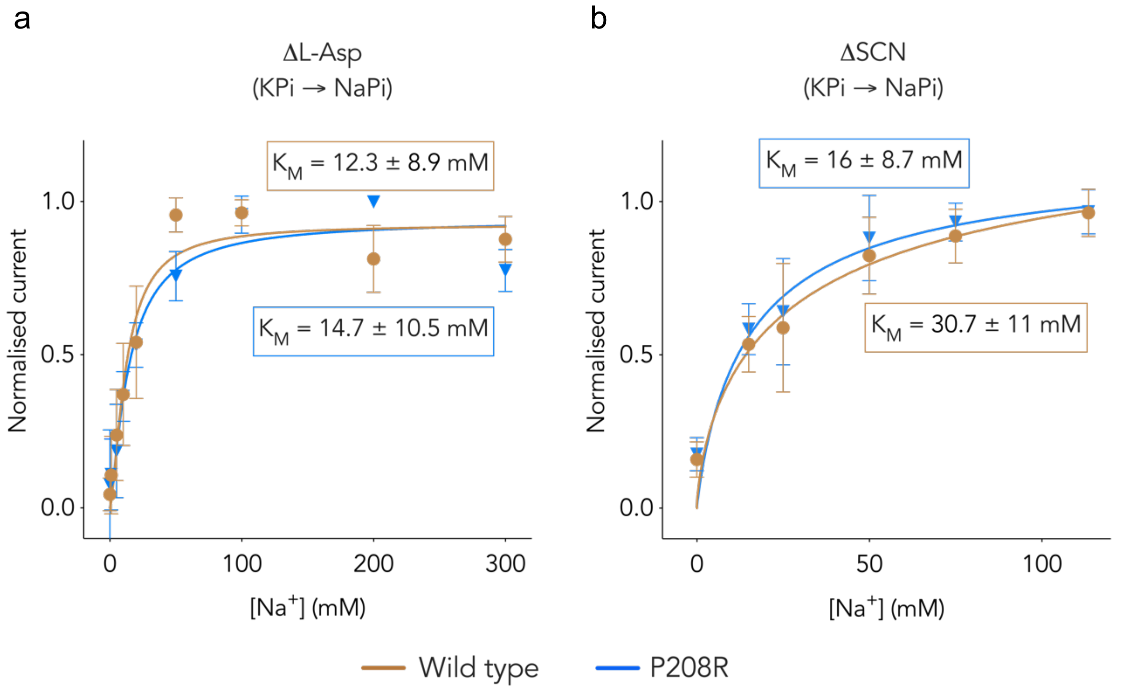

Supplementary figure 2. (a) Sodium dependence of transport of 100 μM L-Asp in wild type and P208R mutant measured with SSM. The plots of the normalized peak currents were fitted using the Hills model with n=1.5 ± 0.4 (WT) and 1.3 ± 0.5 (P208R). The error bars are representative of two biological and two technical replicates; (b) Sodium dependence of transport of 10 mM SCN^- in wild type and P208R mutant measured with SSM. The plots of the normalized peak currents were fitted using the Hills model with n=0.7 ± 0.3 (WT) and 0.9 ± 0.4. The error bars are representative of the standard deviation calculated on two biological and two technical replicates.

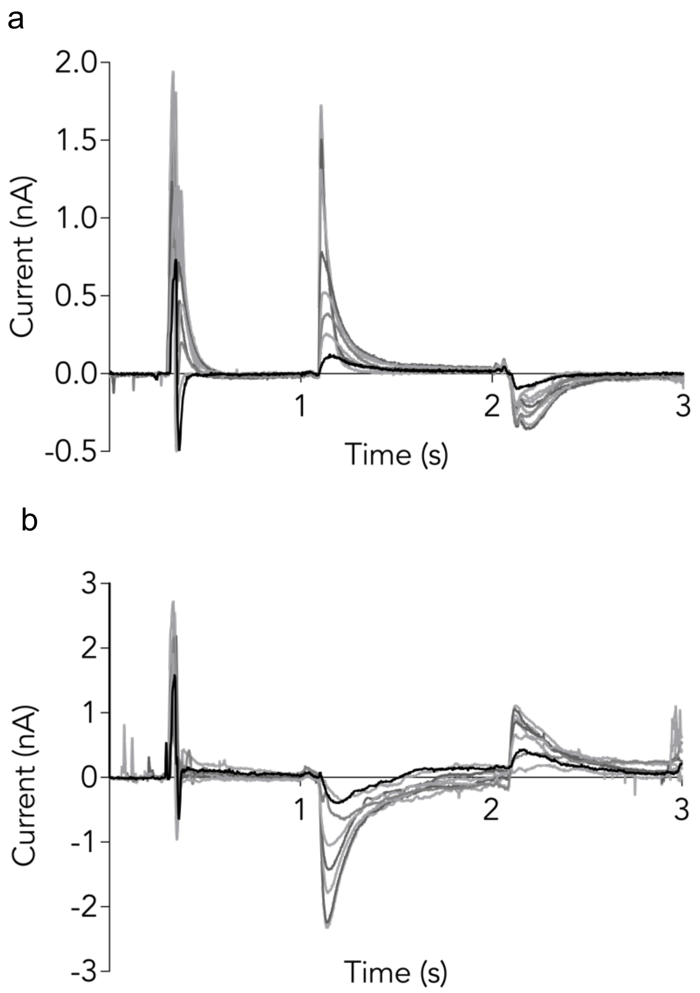

Supplementary figure 3. Representative of full traces of DSE experiments using increasing concentrations of Na^+ on proteoliposomes containing wild type Glt_{Tk}. Na^+-dependence of the transient current measured upon L-Asp jumps (a) or SCN jumps (b). The experiment is explained in full details in Figure 2 of the main text.

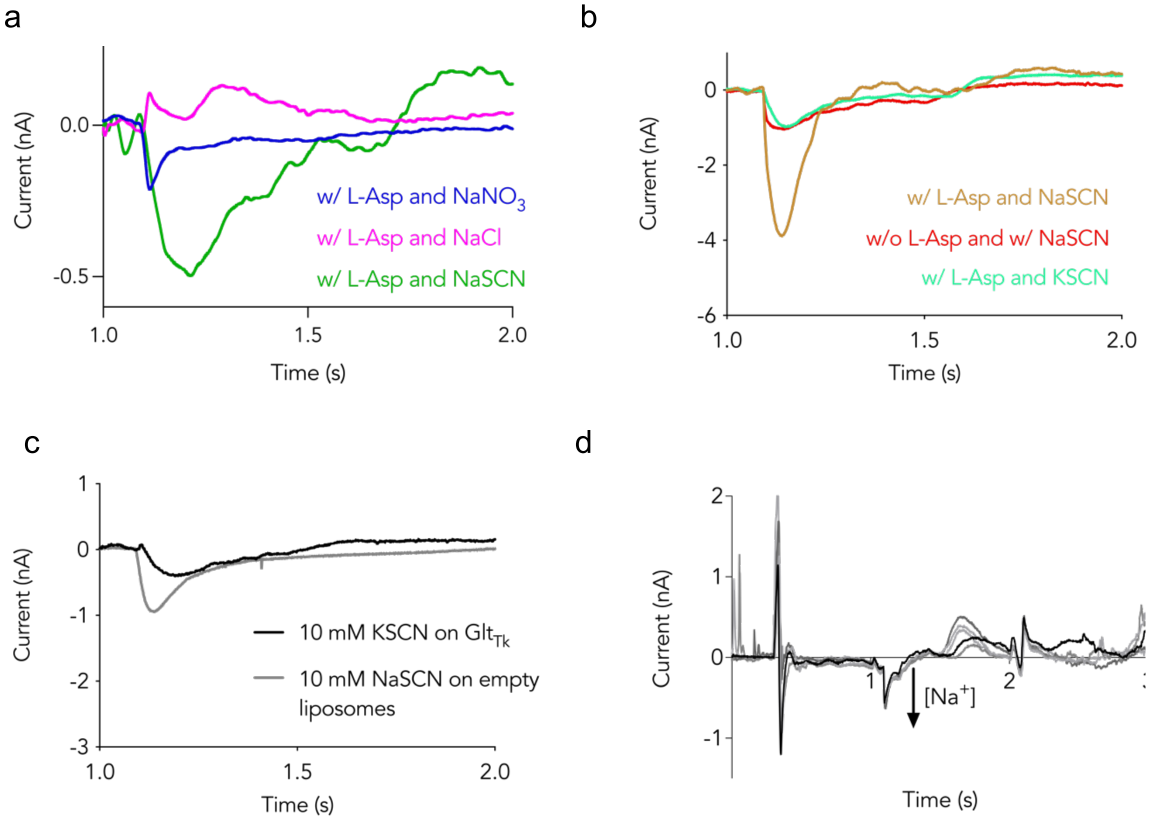

Supplementary figure 4. (a) Comparison of the current amplitudes recorded for wild type Glt_{Tk} upon jumps of 1 mM of three different anions: nitrate, chloride and thiocyanate. : the proteoliposomes are subjected to a change from the resting solution (113.5 mM KPi pH 7 + 100 µM L-Asp) to the non-activating solution (113.5 mM NaPi pH 7 + 100 µM L-Asp) to the activating solution (113.5 mM NaPi pH 7 + 100 µM L-Asp + 1 mM anion) where the signal is measured; (b) Current amplitudes recorded for wild type Glt_{Tk} upon jumps of 10 mM SCN^- in presence or absence of 100 µM L-Asp and 10 mM Na^+ (in the latter case, Na^+ is replaced with K^+); (c) Artefact measured upon 10 mM SCN^- on empty liposomes (in the presence of Na^+ and L-Asp) compared to the signal obtained injecting 10 mM SCN^- on proteoliposomes containing wild type Glt_{Tk} but in absence of Na^+; (d) Artefact of 10 mM SCN^- on empty liposomes upon increasing concentrations of Na^+.

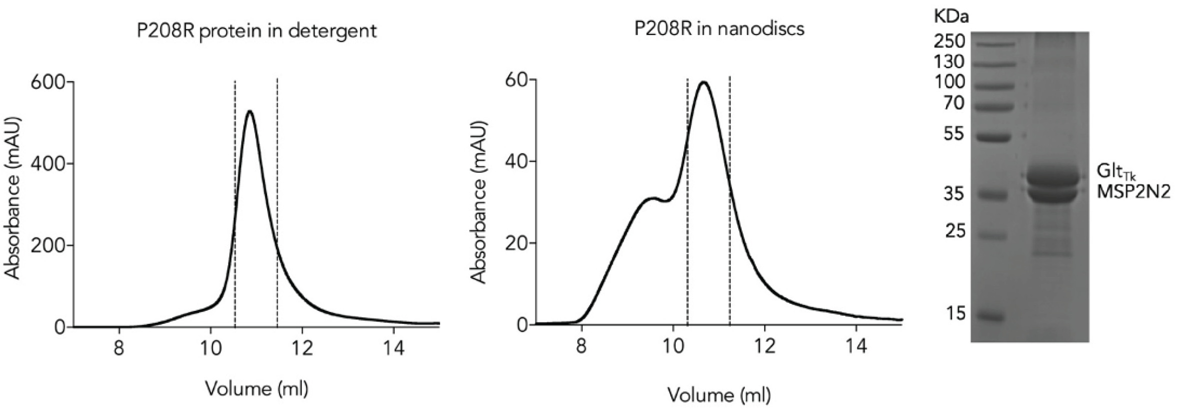

Supplementary figure 5. Quality control of stability of the proteins both in detergent and in nanodiscs. The obtained profiles are fully reproducible as was observed from >10 independent purifications.

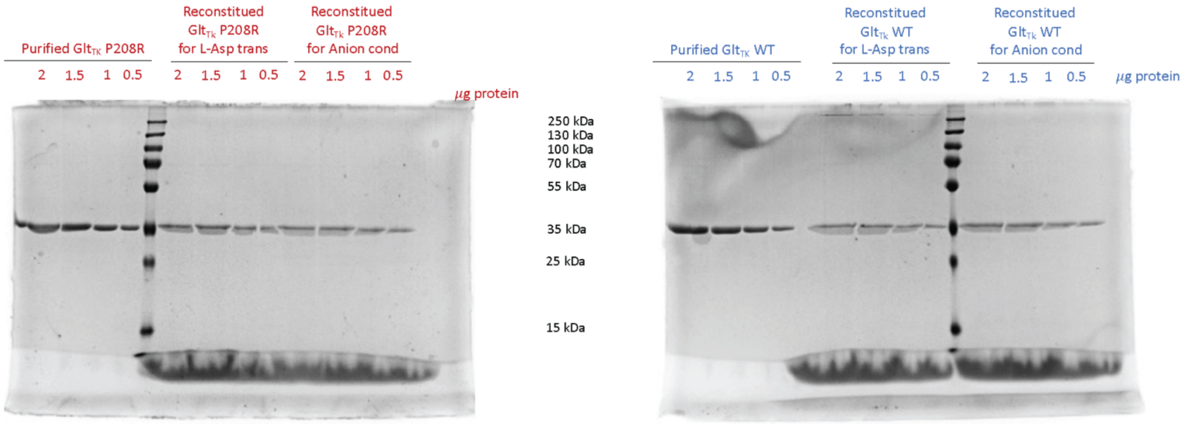

Supplementary figure 6. Estimation of the concentration of reconstituted proteins (WT and P208R) in proteoliposomes for L-Asp transport and anion conductance measurements shown in main text Fig. 3 and Supplementary Fig. 2. Purified Glt_{Tk} in detergent solution was loaded onto the gels as standard for concentration determination by Coomassie blue staining. Reconstitution efficiencies are the same for WT and mutant protein.

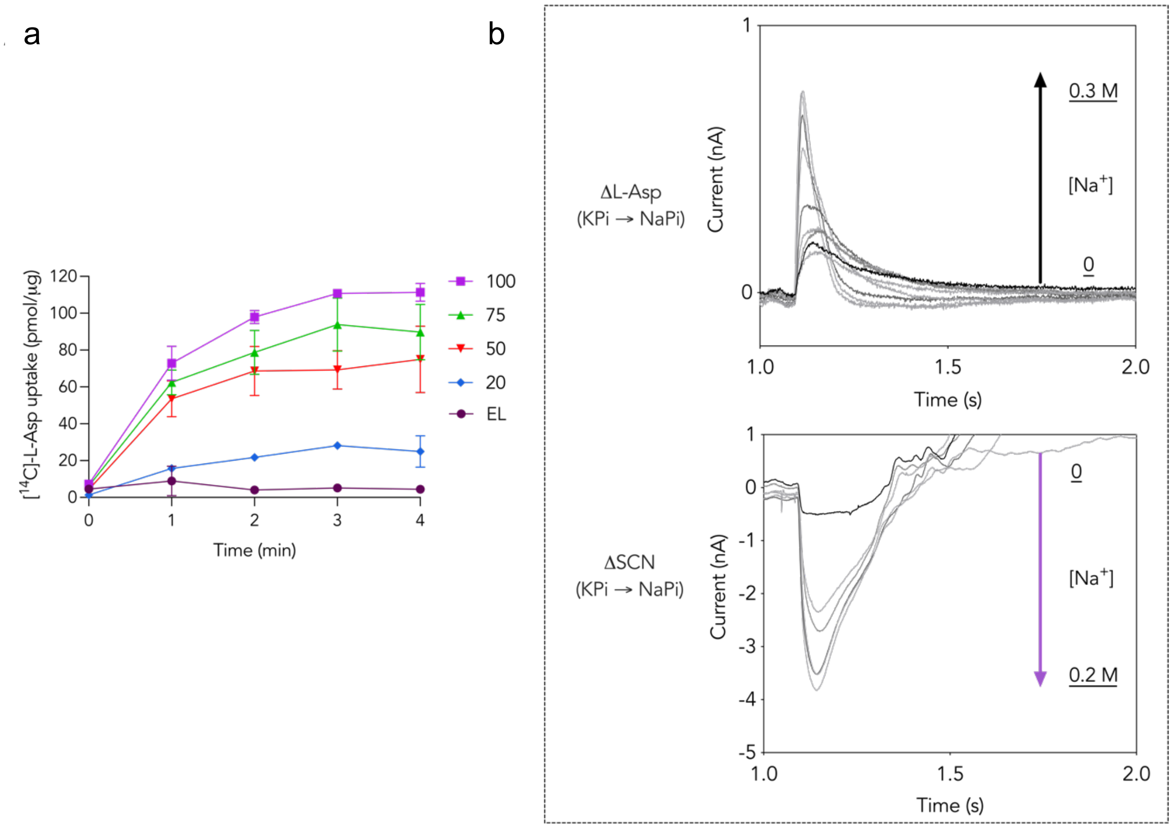

Supplementary figure 7. (a) Time course [14C]-L-Asp uptakes of Glt_{Tk} proteoliposomes reconstituted at different LPRs, the error bars are representative of the standard deviation calculated on three biological replicates; (b - top) The progressive increase of the current amplitude recorded for Glt_{Tk}_P208R upon L-Asp jump in the presence of increasing concentrations of Na^+ in the activating buffer (see Fig. 2 for the details about the experiment); (b – bottom) The progressive increase of the negative current amplitude on Glt_{Tk}_P208R upon SCN^- jump in the presence of increasing concentrations of Na^+ in the activating buffer.

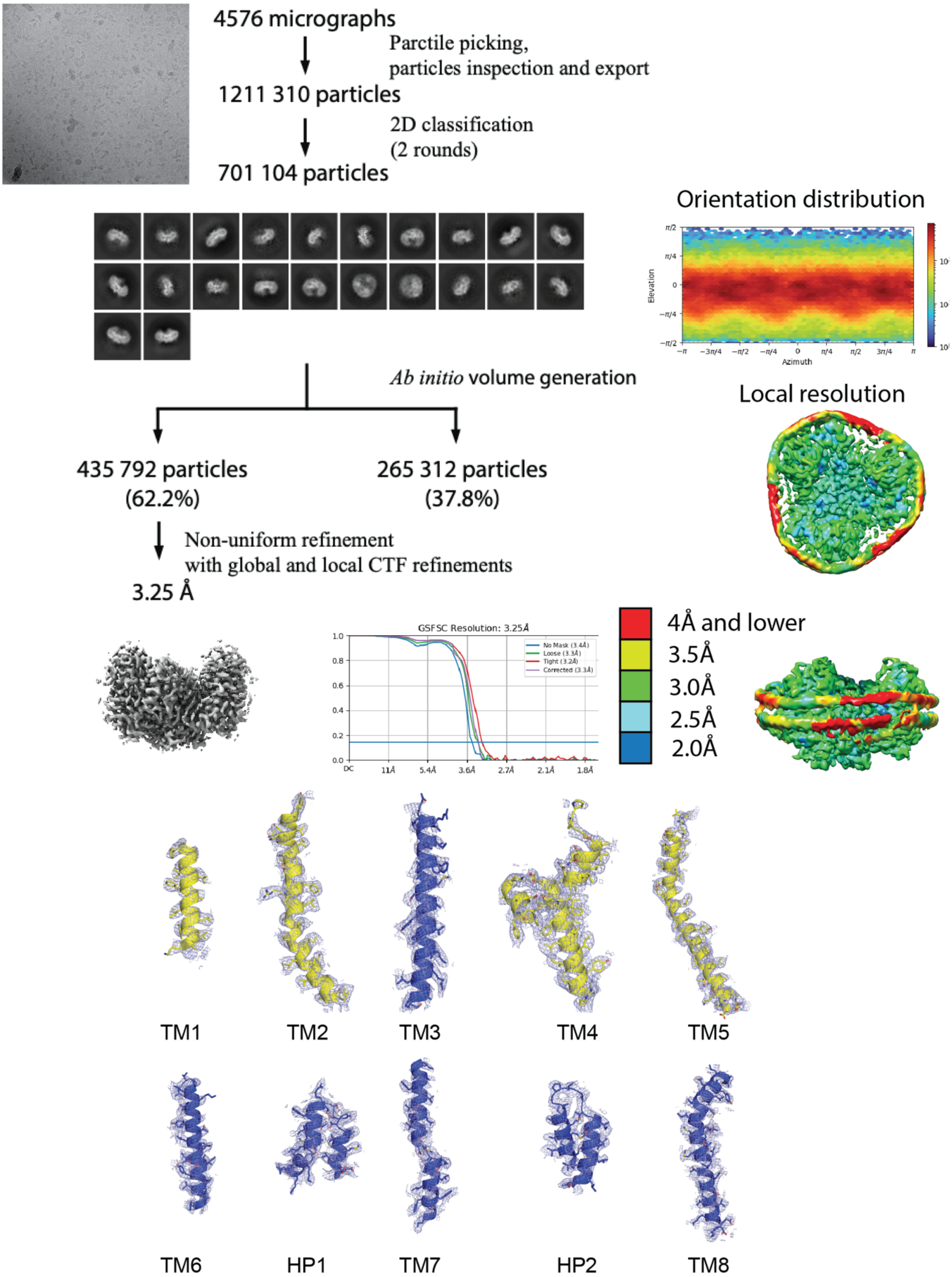

Supplementary figure 8. Workflow for solving the structure of Glt_{Tk}_P208R in nanodiscs using CRYOSPARC. Overall resolution, local resolution and orientation distribution as well as densities for TM1-8 and HP1-2 are shown.

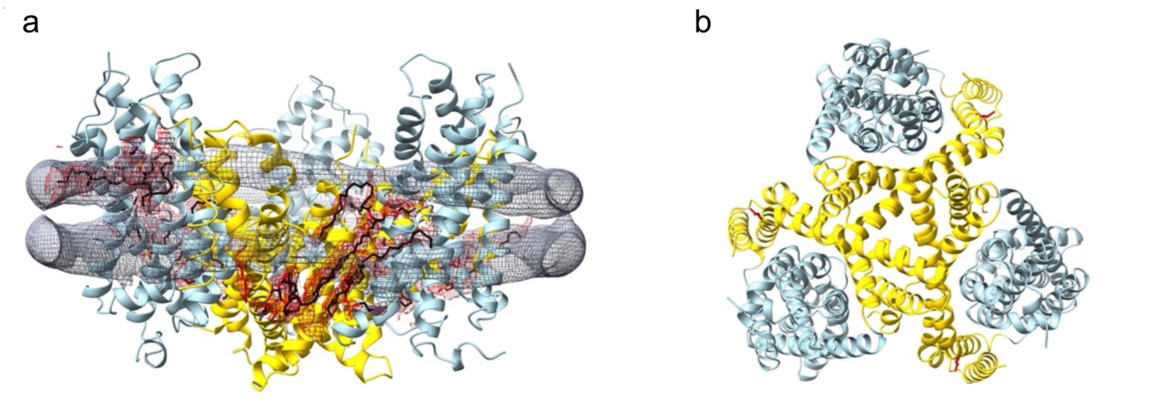

Supplementary figure 9. Trimeric assembly of Glt_{Tk}_P208R in nanodisc obtained at 3.25 Å in outward-occluded facing state. (a) Side view with highlighted phospholipid densities, (b) Top view with highlighted arginine 208 in red stick. EM densities at 3.5σ for nanodisc and putative lipids shown in grey and red respectively.

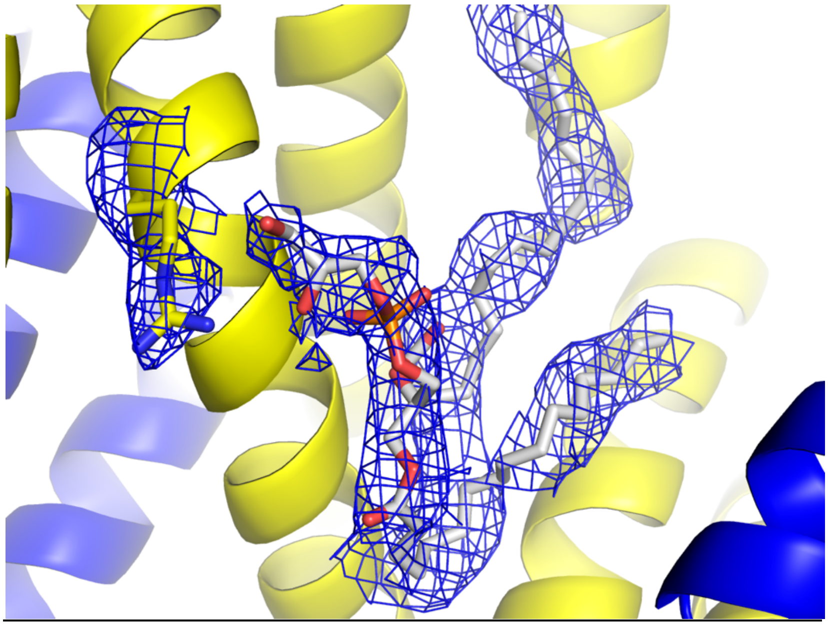

Supplementary figure 10. Arg208 (sticks) of TM5 (yellow) in P208R is pointed towards the lipids of bilayer. The EM density is shown in blue mesh at 4σ. Note that it is not possible to assign the exact phospholipid, hence a molecule of POPG was placed in the density for illustration only.

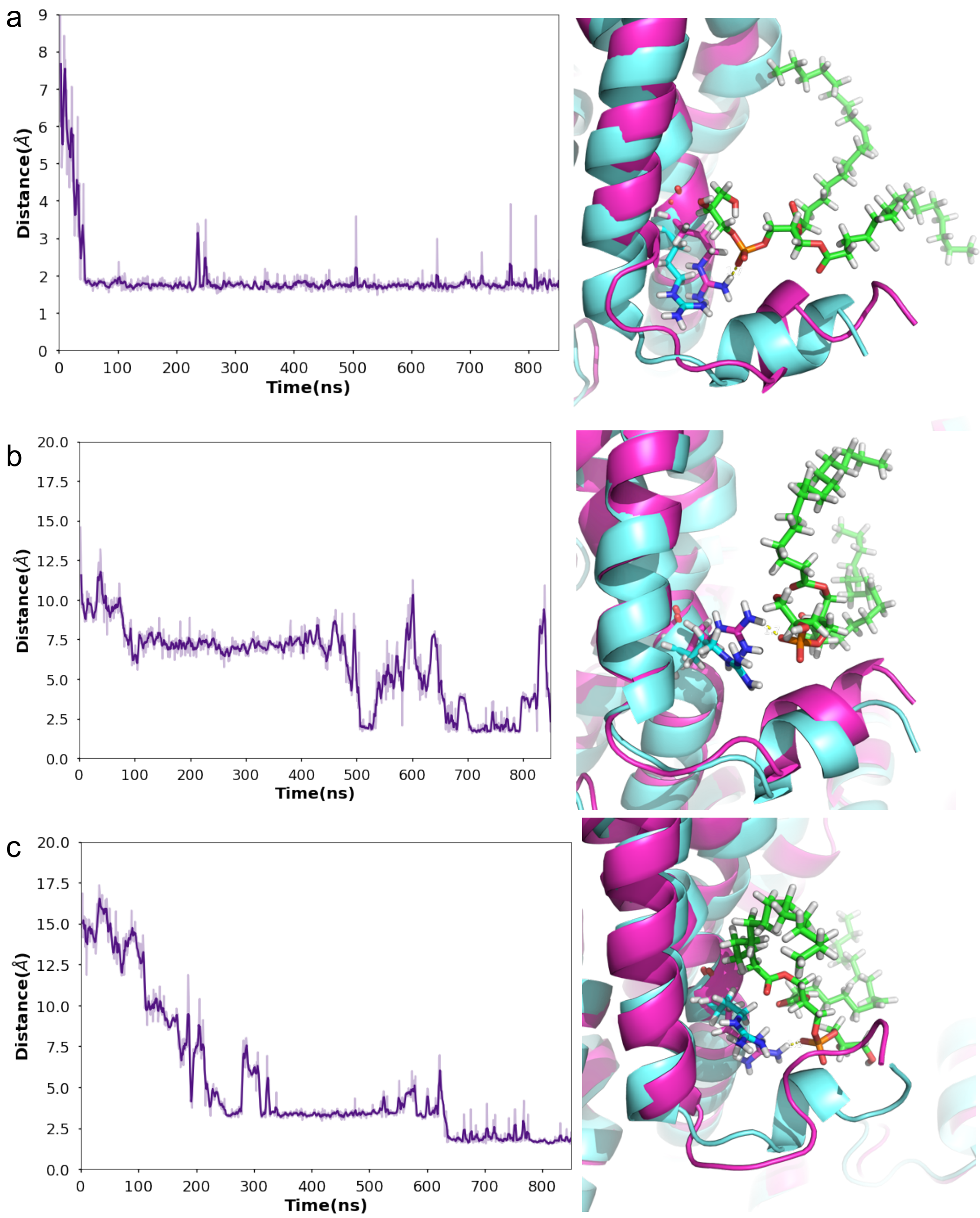

Supplementary figure 11. Formation of protein-lipids interactions and the time evolution of the distance between Arg208 and POPG for each monomer (a-c) in the P208R mutant. Superposition of the start (cyan) and the simulated (violet) models of the P208R mutant (right), with relevant residues represented as sticks. Each calculation is an average of three randomly started calculations.

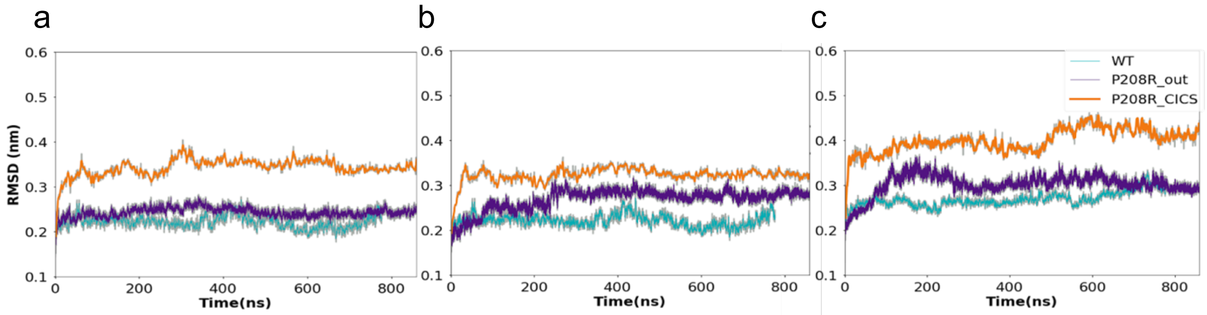

Supplementary figure 12. Backbone RMSD values of the Glt_{Tk} for all monomers (a-c) for the wild type (WT, cyan), P208R mutant in anion conducting state (P208R_in, orange) and inward-inward-outward (P208R_out, violet). Each calculation is an average of three randomly started calculations.

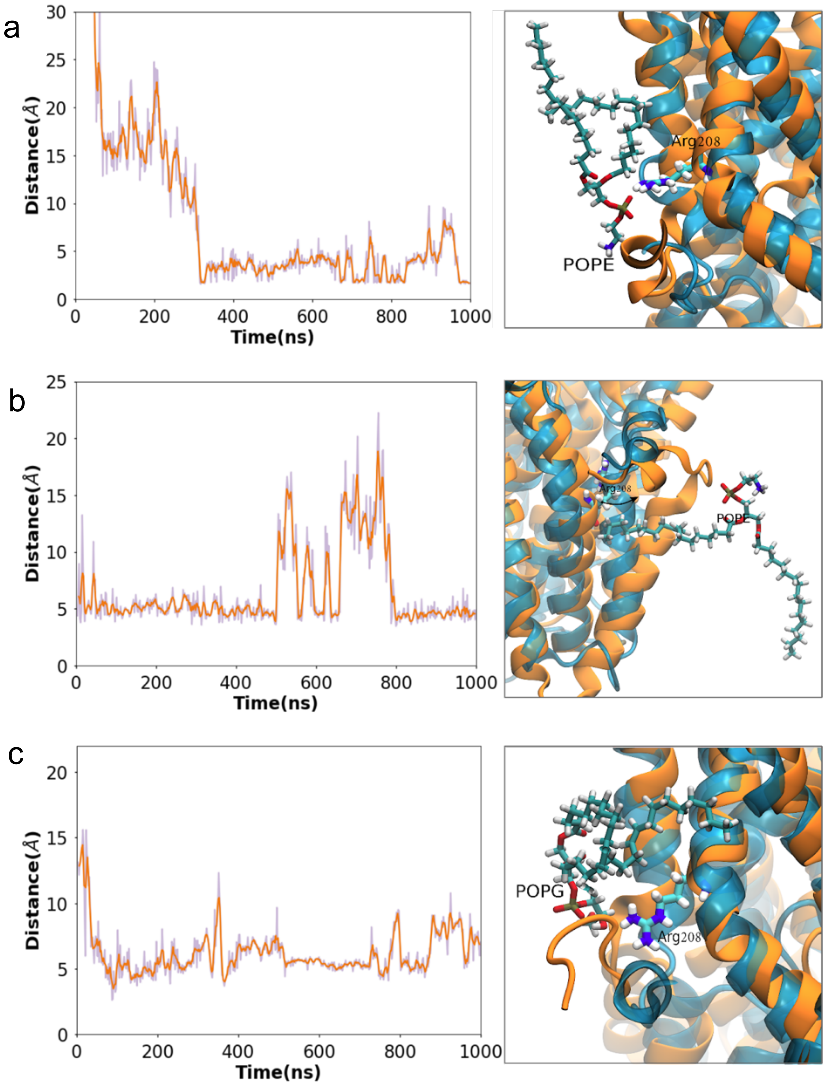

Supplementary figure 13. The threaded model of Glt_{Tk} P208R mutant in the anion conducting state with the time evolution of the distance between Arg208 and POPG for each monomer (a-c). Superposition of the start (cyan) and the simulated (orange) models of the P208R mutant (right), with relevant residues represented as sticks.

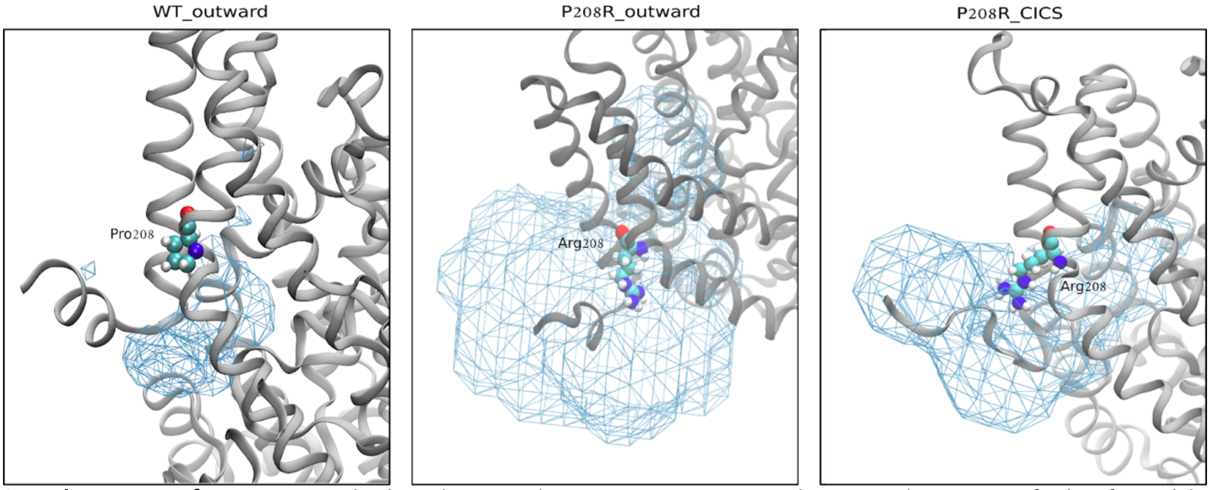

Supplementary figure 14. Calculated water density map (cyan mesh) around Arg208 of Glt_{Tk} for wild type (left) and P208R mutants in outward facing and anion conducting states (middle and right panels) obtained from all-atom MD simulations. Pro208 and Arg208 are shown in VdW representation.
