# Supplementary material for: Mutation in glutamate transporter homologue GltTk provides insights into pathologic mechanism of episodic ataxia 6
Source: Nat Commun. 2023 Mar 31;14:1799. doi: 10.1038/s41467-023-37503-y (PMC10066184; doi:10.1038/s41467-023-37503-y)
Supplement: Supplementary file 3 — Description of Additional Supplementary Files [file 41467_2023_37503_MOESM3_ESM.pdf]

File name: Supplementary Movie 1

Description: Pore widening as seen in MD simulations on P208R mutant. Start (cyan) and the simulated (orange) models are shown. Arg208 shown in vdW representation.
